# Exposure risks and ineffectiveness of total release foggers (TRFs) used for cockroach control in residential settings

**DOI:** 10.1186/s12889-018-6371-z

**Published:** 2019-01-28

**Authors:** Zachary C. DeVries, Richard G. Santangelo, Jonathan Crissman, Russell Mick, Coby Schal

**Affiliations:** 1 Department of Entomology and Plant Pathology, North Carolina State University, Raleigh, NC USA; 2 Center for Human Health and the Environment, North Carolina State University, Raleigh, NC USA; 3 W.M. Keck Center for Behavioral Biology, North Carolina State University, Raleigh, NC USA; 4 Department of Biological Sciences, Columbia University, New York, USA

**Keywords:** Bug bomb, German cockroach, Pesticide residues, Pesticide exposure, Pyrethroids, Total release aerosols

## Abstract

**Background:**

The German cockroach, *Blattella germanica*, is one of the most challenging pests to eradicate from indoor environments. Professional pest control is often prohibitively expensive, prompting low-income residents to turn to over-the-counter consumer products, including total release foggers (TRFs, “bug bombs”). Despite their widespread use, little is known regarding either the associated pesticide exposure risks or the efficacy of TRFs.

**Methods:**

Cockroach-infested homes were recruited into the study. Wipe samples were collected from various surfaces before TRFs were discharged, immediately after, and one month later to determine pesticide exposure risks in 20 homes (divided equally among four different TRF products). Simultaneously, cockroach populations were monitored in all homes to assess the efficacy of TRFs. In parallel, 10 homes were treated with gel baits (divided equally between two bait products), to compare TRFs to a more targeted, low-risk, do-it-yourself intervention strategy.

**Results:**

TRFs failed to reduce cockroach populations, whereas similarly priced gel baits caused significant declines in the cockroach populations. Use of TRFs resulted in significant pesticide deposits throughout the kitchen. Across all products, pesticides, and horizontal kitchen surfaces, pesticide residues following TRF discharge were 603-times (SEM ±184) higher than baseline, with a median increase of 85 times.

**Conclusions:**

The high risks of pesticide exposure associated with TRFs combined with their ineffectiveness in controlling German cockroach infestations call into question their utility in the marketplace, especially because similarly priced and much safer bait products are highly effective in the indoor environment.

## Background

In the United States alone, 82 million households used insecticides in 2012 and $2.65 billion were spent in the “home and garden sector”, representing 50% of all expenditures on insecticides [1]. One of the most prominent pests targeted with insecticides is the German cockroach (*Blattella germanica*). There are many reasons for eliminating indoor cockroach infestations, but primary among them is the central role that cockroaches play as etiological agents in allergic disease and asthma [2]. Allergens produced by German cockroaches can trigger allergies and asthma in sensitized individuals, and the National Cooperative Inner-City Asthma Study found that asthma morbidity was highest in children that experienced both a positive skin-test response and high exposure to cockroach allergens [3]. The National Survey of Lead and Allergens in Housing, a nationwide survey conducted by the U.S. National Institute of Environmental Health Sciences (NIEHS) and the U.S. Department for Housing and Urban Development (HUD), found detectable levels of the cockroach allergen Bla g 1 in 63% of homes [4], with higher concentrations in high-rise apartments, urban settings, older homes, and low-income households [4, 5]. Moreover, because cockroaches move freely between waste and food, they can acquire, carry, and disseminate pathogenic bacteria, helminths, fungi, protozoa, and viruses in their digestive system [6]. Thus, the persistence of cockroaches in homes poses significant health risks to humans.

Indoor cockroach infestations are often targeted with residual liquid or aerosol sprays that contain broad-spectrum insecticides, most commonly pyrethroids [7]. However, high levels of resistance to pyrethroids and their repellency to cockroaches severely compromise the efficacy of most residual sprays [7]. Moreover, these products can deposit considerable insecticide residues throughout the home [8]. Environmental data collected by the U.S. Environmental Protection Agency (EPA) and HUD on a stratified, nationally representative sample of 1131 residences found extensive pesticide residues in homes [9]. Despite the continual use of residual sprays, insecticides formulated as baits offer more effective and safer alternatives in cockroach interventions [10, 11].

Because professional pest control interventions can be prohibitively expensive, consumer-based pesticide products are commonly used in do-it-yourself (DIY) pest control, especially in low-income homes. Total-release foggers (TRFs) are often deployed as spatial insecticides, designed to fill a room with fine particles of aerosolized insecticide. They are considered by consumers to be highly effective against all pests (as the common name “bug bomb” implies). TRFs generally contain toxicity category III (based on acute toxicity) active ingredients (pyrethrins and pyrethroids), various synergists meant to inhibit microsomal detoxification by insects, and aerosol propellants that are often flammable. These products are responsible for substantial acute and chronic health effects, explosions and fires, and persistent environmental contamination indoors. These effects were first characterized by a 2008 US Centers for Disease Control and Prevention (CDC) report that summarized 466 fogger exposures in eight States over a five-year period, documenting respiratory, gastrointestinal, neurological, ocular, dermatologic, and cardiovascular adverse symptoms [12]. A similar summary from Texas, USA documented 2855 fogger exposures over an 8-year period [13]. Despite these reports, the magnitude of health, economic, and environmental damage is poorly documented, and likely underestimated. Indeed, a follow-up report from the New York City Department of Health and Mental Hygiene, USA [14] stated that the 2008 CDC report understated reported exposures. This follow-up report also showed that health effects are much more likely to occur from exposures to TRFs than from other pesticide formulations, and moderate or major health effects were more than twice as likely to occur from TRF exposures as from all pesticides, and seven times as likely as from rodenticides. While many of the fogger-associated illnesses and injuries result from inadvertent exposures during their deployment (leaving the premises too late, re-entering too soon, discharging too many foggers, failing to notify others), studies suggest that TRFs deposit large amounts of insecticides in areas easily accessible to humans, especially small children [8, 15]. The residual pyrethroids on household surfaces can exacerbate a number of chronic health conditions [16], although the health effects from chronic exposure are still under debate.

TRF products appear to contribute significantly to the disproportional pesticide exposure already documented for those living in affordable housing [17, 18]. The report from New York City’s Department of Health and Mental Hygiene [14] contends that “the health risks associated with the use of foggers are not justified given their likely poor efficacy”. Recently, Jones and Bryant [19] showed that over-the-counter TRFs were indeed ineffective at controlling bed bug infestations. Surprisingly however, there are no reports on the relative efficacy of modern TRF products against their primary target, the German cockroach. Therefore, we designed a study to assess the efficacy and exposure risks of TRFs in cockroach-infested homes.

## Methods

### Ethics statement

The North Carolina State University Institutional Review Board (IRB) approved this study (#1459). Before participation, adult participants (> 21 yrs. old) provided written informed consent. Demographic data on participants were not gathered in this study, as we were interested in a cockroach intervention and the environmental outcomes in cockroach-infested residences, independent of the demography of the residents.

### Recruitment of participants

Apartments in five low-income communities within the city of Raleigh NC, U.S.A., were visited and residents were queried regarding cockroach infestations. Apartments were in multi-unit low-rise buildings, duplexes, and row homes. Residents were first informed of the purpose of the study, provided informed consent, then asked if (a) they had seen any live cockroaches, and (b) if they were interested in participating in the study. If the resident reported seeing cockroaches and agreed to participate, the home was visually inspected for the presence of cockroaches. If the home was expected to qualify based on sufficient numbers of live German cockroaches or evidence of cockroaches, the home was recruited into the study. Official enrollment followed standard cockroach population quantification, implemented through trapping (see “Intervention effectiveness – Assessment of relative cockroach population size” below).

### Interventions

Four different TRF products were used, representing several insecticide active ingredients and manufacturers: Hot Shot No-Mess Fogger_2_ with Odor Neutralizer (Hot Shot 2; 85 g, 0.333% tetramethrin, 0.834% permethrin, 1.667% piperonyl butoxide; Spectrum Group-United Industries, St. Louis, MO, U.S.A.), Hot Shot No-Mess Fogger_3_ with Odor Neutralizer (Hot Shot 3; 170 g, 0.200% tetramethrin, 0.860% cypermethrin, 0.500% piperonyl butoxide; Spectrum Group-United Industries), Raid Max Concentrated Deep Reach Fogger (Raid Deep; 60 g, 1.716% cypermethrin; SC Johnson, Racine, WI, U.S.A.), and Raid Fumigator (10 g, 12.600% permethrin; SC Johnson). Five replicate homes were treated with each TRF product, one home in each of five apartment complexes (20 TRF-treated homes).

Each TRF was discharged in the kitchen following the product label instructions and EPA precautions (https://www.epa.gov/safepestcontrol/safety-precautions-total-release-foggers; last accessed April 15, 2017). Briefly, all residents vacated the apartments for 4–6 h, windows and doors were closed, air conditioning and gas stove pilot lights were turned off, cabinet doors were opened and contents as well as immovable kitchen appliances were covered with newspapers, and aquaria were moved out of the kitchen. Four to six hours later, the apartment was ventilated, newspapers discarded, dishes rinsed, and residents allowed to re-enter.

Running in parallel, 10 additional apartments were treated with only gel baits. Five homes, one in each apartment complex, were treated with a consumer bait, Combat Gel Bait (0.010% fipronil; Combat Insect Control Systems-The Dial Corporation, Scottsdale, AZ, U.S.A.), and another set of five homes, one in each apartment complex, received a professional bait, Maxforce Gel Bait (0.010% fipronil; Bayer Environmental Science, Robinson Township, PA, U.S.A.). Bait was dispensed as needed at each of three visits (baseline, two weeks, one month). At the conclusion of the study, all TRF-treated apartments were provided thorough gel bait interventions.

### Intervention effectiveness – Assessment of relative cockroach population size

At baseline, and subsequently two and four weeks after treatment, six glue-board sticky-traps (Victor Roach Pheromone Trap, Woodstream Corporation, Lititz, PA, U.S.A.) were placed in kitchen locations where cockroaches commonly aggregate. The traps were collected the following day and enumerated in the lab. Changes in each cockroach population (apartment) were assessed relative to the baseline trap catch. Homes were enrolled in the study if at least 50 cockroaches were trapped at baseline.

### TRF efficacy – Caged sentinel cockroaches

After enrollment, cockroaches were collected from the kitchen using a modified Eureka Mighty-Mite 7.0-A vacuum cleaner (Eureka Company, Charlotte, NC, U.S.A.). Live cockroaches were collected into a mesh-lined plastic tube attached to the distal end of the vacuum’s extension tube. Apartment-collected male cockroaches were used as caged sentinels for determining product efficacy in the same apartment where they were collected. Prior to discharging the TRF, 40 laboratory raised, insecticide-susceptible adult male cockroaches and 40 home-specific apartment-collected males were placed into the home as sentinels. Twenty cockroaches from both the laboratory population and the apartment-specific population were placed in two uncovered cages on the floor 1.0 m away from the TRF (referred to as “floor”), and the other 20 cockroaches from each population were placed in two uncovered cages in an upper cabinet (lowest shelf, referred to as “upper cabinet”). The inside walls of the cages were coated with petroleum jelly to prevent cockroaches from escaping. Four to six hours after the TRF was discharged, and it was safe to re-enter the apartment, the sentinel cockroaches were collected, returned to the laboratory, transferred to a clean cage, and assessed for mortality 24 h later.

### Pesticide residue analysis

Kitchens were sampled for insecticide residues at three time points during the study: before TRF use (baseline), immediately (4–6 h) after TRF discharge, and one month later. Areas sampled included the floor at both 0.5 m and 1.0 m from the site of the TRF, the nearest countertop to the TRF (~ 0.9 m high), the inside (base) of an upper level cabinet (~ 1.4 m), and the nearest wall to the TRF at a height of 0.9 m (representing the height of a child). The same areas of the kitchen, but not the same spots, were sampled at each subsequent visit. Samples were collected by wiping an area of 100 cm^2^ with a cotton swab wetted with isopropyl alcohol for 1 min. Each swab sample was placed into a 20 ml glass vial, immediately returned to the laboratory and stored at − 30 °C until extraction.

Swab samples were analyzed for the specific active ingredients used in the TRF products, which included permethrin (sum of *cis-* and *trans-* isomers), cypermethrin (sum of all isomers), tetramethrin (sum of all isomers), and PBO (pyrethroid synergist). Additionally, swab samples were analyzed for fipronil residues (active ingredient from baits used). Each sample was fortified with 500 ng of the surrogate recovery standard (SRS) ^13^C_6_-*trans*-permethrin (Cambridge Isotope Laboratories Inc., Tewksbury, MA, U.S.A.). Samples were extracted and sonicated twice with ethyl acetate. Solvent volume was then reduced, and samples were cleaned using a 3 ml prefabricated solid phase extraction (SPE) column containing 500 mg of silica gel (Supelclean LC-Si SPE Tube, Sigma Aldrich, St. Louis, MO, U.S.A.). The SPE column was conditioned with 5 ml of hexane, the sample was loaded onto the column and eluted with 5 ml of 50% ether in hexane. Each eluted sample was spiked with 500 ng of the internal standard (IS) 4,4′-dibromobiphenyl (DBBP, AccuStandard Inc., New Haven, CT, U.S.A.), evaporated to near dryness under nitrogen, resuspended in 1 ml of hexane, and stored at − 30 °C until analysis.

Samples were analyzed using an Agilent Technologies 6890 GC coupled to an Agilent 5975 mass spectrometer (GC-MS). The GC was equipped with a 30 m × 0.25 mm × 0.25 μm (5%-phenyl)-methylpolysiloxane Agilent J&W HP-5 ms column preceded by a 3 m deactivated guard column. The temperature program was: 100 °C for 1 min, then 5 °C/min to 225 °C, then 2 °C/min to 256 °C, then 10 °C/min to 320 °C where it was held for 10 min. Mass spectrometry conditions were: transfer line at 280 °C, ionization source at 230 °C, and quadrupole at 150 °C. One quantification ion was used for each pesticide (Table 1). Ten calibration curve solutions ranging from 0.1 μg/ml to 100 μg/ml for all TRF insecticides (Sigma-Aldrich) were used to generate calibration curves via log-transformed linear regression. Extracted samples were corrected for both the SRS and IS and quantified using the calibration curve. Each calibration curve solution was run a minimum of three times, interspersed evenly among field-collected samples. If any compound exceeded the upper point in the calibration curve by more than 15%, the sample was diluted and re-analyzed. Method detection limits (MDLs) were determined using the guidelines from 40 CFR Part 136, Appendix B.

**Table 1:** Retention times, quantification ions, and qualification ions for insecticide residue analyses by GC-MS.

Insecticide	Retention time (min)	Quantification ion	Qualifying ion
Fipronil	23.00	367	213
Piperonyl butoxide	28.90	176	149
Tetramethrin	29.95–30.30	164	123
Permethrin (*cis*- and *trans*-)	35.35–35.80	183	163
Cypermethrin	38.50–39.40	163	181

### Data analyses

Two-way repeated measures ANOVA was used to compare changes in cockroach population levels over time (baseline, two weeks, four weeks) among different treatments (TRFs and baits). Due to interactive effects, repeated measures ANOVA was used to evaluate changes in cockroach population within each treatment, with means at different times compared using the Tukey-Kramer multiple comparison test.

Three-way ANOVA was used to compare sentinel cockroach percent mortality (arcsine square root transformed) among TRF products, population (laboratory-raised or apartment-collected), and location (floor or upper cabinet).

The effects of each TRF treatment on insecticide residues were evaluated using repeated measures ANOVA (within each treatment) on log-transformed values. Treatments were defined by the insecticide(s) in each TRF product and swab locations quantified over time, with means at 4–6 h and one month after the TRF intervention compared to the baseline mean using Dunnett’s test. Prior to log-transformation, all values had the respective insecticide MDL added to them. Comparisons were also made among swab locations for each TRF treatment 4–6 h after discharge using ANOVA. Insecticide residues were evaluated only for the Combat gel bait group, and not the Maxforce bait group, at baseline and one month. Additionally, three apartments were removed from the study for either failing to complete the study or not following the approved protocol. Also, some samples have missing values due to problems with sample collection or sample analysis (reflected by sample size).

All statistical analyses were performed using SAS 9.4 (SAS Institute, Cary, NC, U.S.A.).

## Results

### Effects of interventions on cockroach populations

**Fig. 1:**
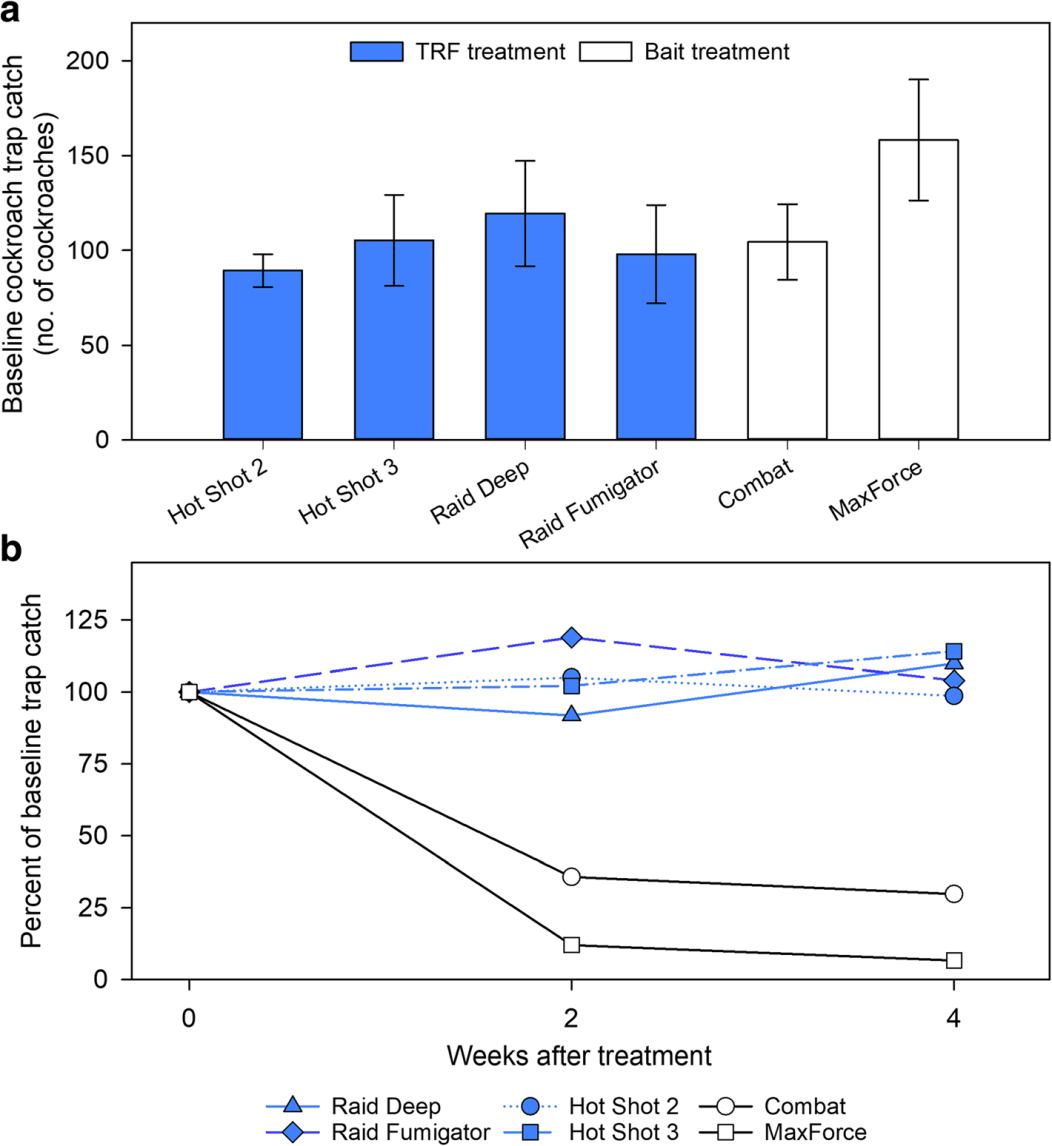
Effects of six interventions (4 TRF products, 2 gel baits) on *B. germanica* populations. Total cockroaches trapped are displayed for each intervention at baseline (**a**), and the percentage of trap catches at 2 and 4 weeks post-intervention relative to baseline (**b**). Error bars represent SEM.

There were no significant differences in the baseline cockroach trap catch, a gauge of population level, among the treatments (*F*_5,22_ = 0.97, *p* = 0.4553) (Fig. 1a). Cockroach trap catch was significantly affected by both treatment and time after intervention (interactive effect); therefore, the effect of each intervention was analyzed separately using repeated measures ANOVA. Only the two bait treatments resulted in significant declines in trap counts relative to baseline (Combat: *F*_2,8_ = 12.40, *p* = 0.0035; Maxforce: *F*_2,8_ = 21.37, *p* = 0.0006) at both two- and four-weeks after the intervention (comparisons of least square means with Bonferroni correction, Fig. 1b). Trap counts in all TRF treatments did not change significantly from baseline counts (*p* > 0.25).

### Responses of sentinel cockroaches to TRFs

**Fig. 2:**
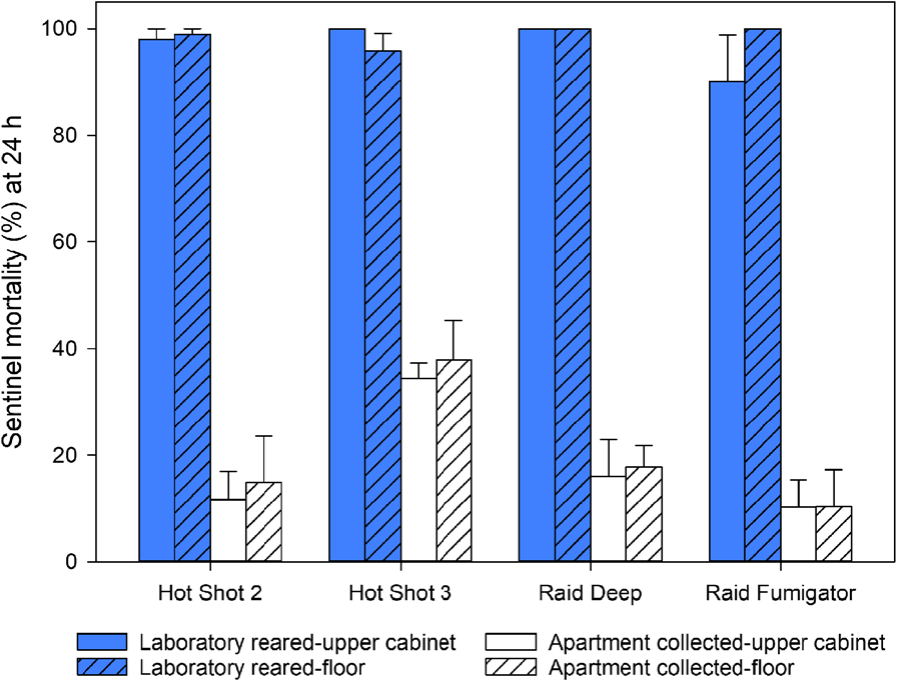
Sentinel cockroach mortality (%) in response to TRF product, population origin (laboratory reared cockroaches are insecticide susceptible, apartment collected cockroaches were collected in each respective home prior to TRF use), and location (upper cabinet, floor). *n* = 5 homes per TRF product; 20 cockroaches per cage

Both the origin of the cockroaches (laboratory or apartments) and the TRF product significantly affected mortality of sentinel cockroaches, while their placement (floor or upper cabinet) had no effect (*F*_15,68_ = 48.95, *p* < 0.0001; Table 2). Mean percent mortality was significantly higher in the insecticide-susceptible laboratory cockroaches than in apartment-collected cockroaches. Despite TRF products having a significant effect on the model, no TRF product provided > 38% mortality in the sentinel cockroaches collected in apartments, with some resulting in < 11% mortality (Fig. 2).

**Table 2:** Effects of cockroach population (laboratory-reared [insecticide-susceptible], apartment-collected), placement (floor, upper cabinet), and TRF product on sentinel cockroach mortality. This includes the interaction terms from three-way ANOVA. Significance indicated by a * (*p* < 0.05)

Source	Type III SS	*d.f.*	*F*	*p*
TRF product	0.61	3	5.37	0.0022*
Cockroach population (laboratory-reared or apartment-collected)	26.8	1	710.76	< 0.0001*
Sentinel cage placement (kitchen floor or upper cabinet)	0.01	1	0.33	0.5698
TRF product x Cockroach population	0.52	3	4.61	0.0054*
Cockroach population x Sentinel cage placement	0.00	1	0.00	0.9978
TRF product x Sentinel cage placement	0.04	3	0.33	0.8071
TRF product x Cockroach population x Sentinel cage placement	0.12	3	1.13	0.3438
Error	2.56	68

### Pesticide residues in kitchens

At baseline, three of the four TRF active ingredients (PBO, permethrin, cypermethrin) were detected in > 86% of the surface swabs (Table 3). Permethrin was detected at the highest frequency (> 91% of all surface swabs), while cypermethrin was detected at the highest average concentration at baseline (> 10 ng/cm^2^ for all swab locations, Table 3). Tetramethrin was detected, but at much lower rates than the other TRF active ingredients (< 38% for all swab locations, Table 3). Fipronil was not detected in any of the baseline samples we swabbed from five different surfaces in the kitchen (0.5 m, 1.0 m, counter, cabinet, wall).

**Table 3:** Insecticide residues at baseline swabbed from five different kitchen surfaces

			Detection frequency (%)^a^	ng/cm^2^						
	Insecticide	N		MDL^b^	Mean^c^	SEM^d^	Median	Max	GM^e^	GSD^f^
0.5 m Floor	Fipronil	21	0	1.077	–	–	<MDL	<MDL	–	–
	PBO^g^	21	100	0.192	3.655	0.959	2.021	17.025	2.018	32.455
	Tetramethrin	21	10	0.133	–	–	<MDL	3.517	–	–
	Permethrin	21	90	0.211	3.001	0.735	1.253	10.939	1.609	33.272
	Cypermethrin	21	86	0.625	10.866	2.496	7.165	47.742	6.290	33.816
1.0 m Floor	Fipronil	22	0	1.077	–	–	<MDL	<MDL	–	–
	PBO	22	100	0.192	3.214	0.693	2.518	12.292	1.959	29.281
	Tetramethrin	22	18	0.133	–	–	<MDL	25.694	–	–
	Permethrin	22	95	0.211	2.840	0.731	1.628	15.629	1.683	29.225
	Cypermethrin	22	95	0.625	10.559	1.559	8.950	24.866	7.945	24.013
Counter	Fipronil	22	0	1.077	–	–	<MDL	<MDL	–	–
	PBO	22	77	0.192	0.749	0.133	0.666	2.746	0.588	21.730
	Tetramethrin	22	14	0.133	–	–	<MDL	18.458	–	–
	Permethrin	22	95	0.211	0.807	0.182	0.497	3.529	0.603	20.309
	Cypermethrin	22	82	0.625	18.726	7.085	5.132	148.070	5.979	49.307
Cabinet	Fipronil	21	0	1.077	–	–	<MDL	<MDL	–	–
	PBO	21	71	0.192	19.587	17.007	1.170	359.082	1.186	68.703
	Tetramethrin	21	38	0.133	–	–	<MDL	232.830	–	–
	Permethrin	21	90	0.211	11.737	3.906	1.645	65.268	2.786	65.997
	Cypermethrin	21	67	0.625	28.370	9.804	6.146	150.993	6.138	76.229
Wall	Fipronil	22	0	1.077	–	–	<MDL	<MDL	–	–
	PBO	22	95	0.192	18.750	11.500	1.562	253.616	2.717	61.764
	Tetramethrin	22	36	0.133	–	–	<MDL	190.180	–	–
	Permethrin	22	86	0.211	36.059	26.211	2.075	576.275	3.089	72.837
	Cypermethrin	22	64	0.625	24.946	8.328	5.833	133.071	5.910	72.920

^a^percentage of samples with residue quantities above the MDL

^b^*MDL* Method Detection Limit

^c^Means were only calculated for those pesticides with > 40% detection frequency

^d^*SEM* Standard Error of the Mean

^e^*GM* Geometric Mean

^f^*GSD* Geometric Standard Deviation

^g^*PBO* Piperonyl butoxide

**Fig. 3:**
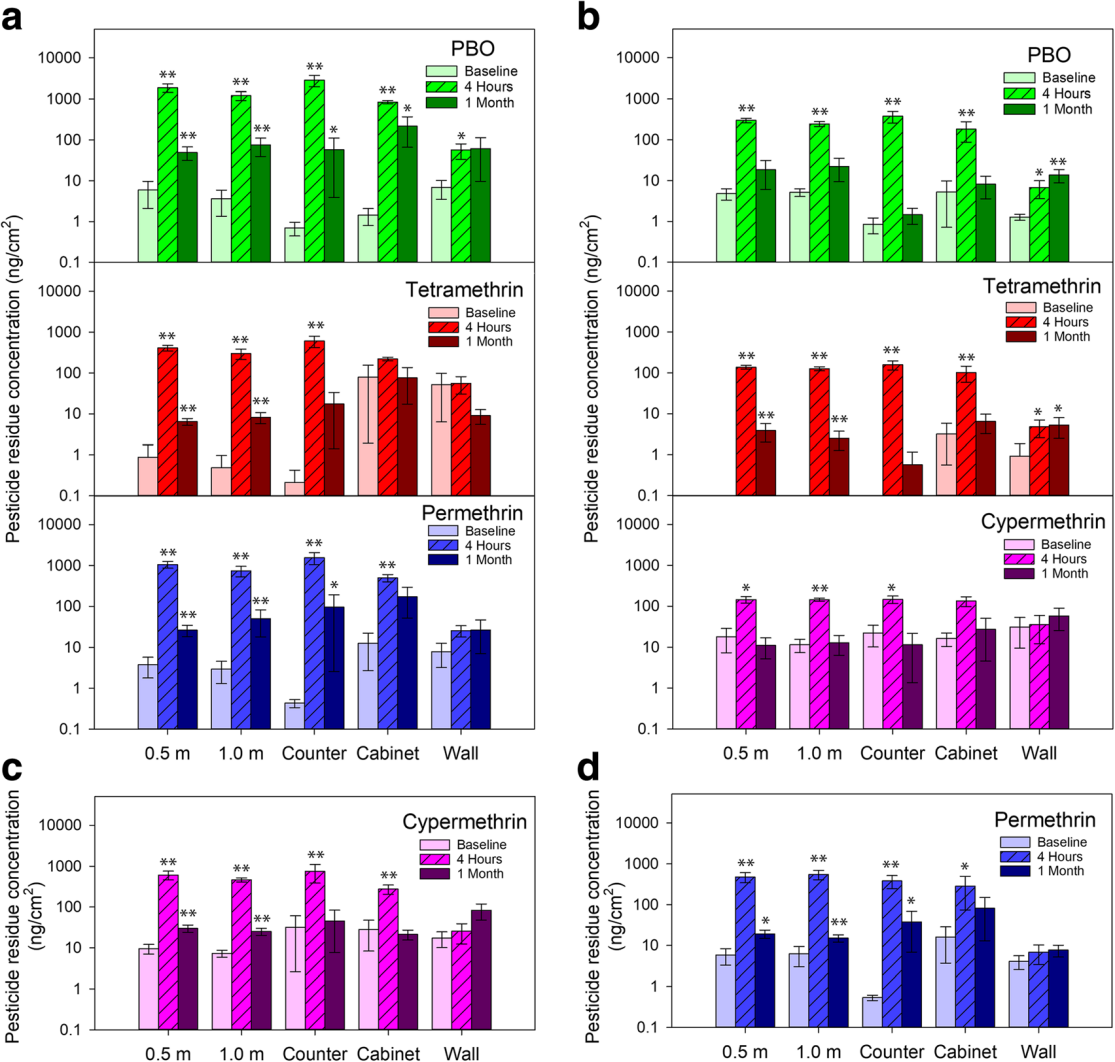
Insecticide residue concentrations in swab samples from homes treated with the following total release foggers (TRFs): **a** Hot Shot No-Mess Fogger_2_ with Odor Neutralizer, **b** Hot Shot No-Mess Fogger_3_ with Odor Neutralizer, **c** Raid Max Concentrated Deep Reach Fogger, and **d** Raid Fumigator. Insecticide residues were measured at baseline, 4 h post-discharge, and 1 month post-TRF intervention. Sites swabbed for insecticide residues included the floor at 0.5 m and 1 m from the TRF discharge site (middle of the kitchen), the nearest countertop to the TRF discharge site, the inside of an upper level cabinet, and the nearest wall to the TRF discharge site at a height of 90 cm from the floor. Error bars represent SEM. Significant differences in insecticide concentrations compared to baseline levels (as determined by Dunnett’s test) are indicated with * (*p *< 0.1) or ** (*p *< 0.05)

Total release foggers deposited significant amounts of insecticide residues on all horizontal kitchen surfaces (Fig. 3). Of the 32 combinations of insecticide by swab location on horizontal surfaces (e.g. PBO at 0.5 m, permethrin on the kitchen counter, etc.), 27 (84%) had significant increases (*p *< 0.05) in residues 4–6 h after the TRF was discharged (94% had moderate increases in insecticide residues, *p* < 0.1). The average increase in insecticide residues on horizontal surfaces 4–6 h after TRF discharge was 603 ± 184 (SEM) times, with a median increase of 85 times relative to baseline. After one month, 34% had significantly higher residues relative to baseline levels (50% had moderate increases).

None of the insecticide by wall sample combinations (vertical surfaces) had significant elevation in pesticide residues 4–6 h after TRF discharge (3 [38%] had moderate increases, Fig. 3). After one month, one (13%) insecticide by wall sample combination had significantly elevated pesticide residues relative to baseline (2 [25%] had moderate increases).

Pesticide residues 4–6 h after TRF discharge varied significantly across sampling locations within the kitchen (*p* < 0.0076 for all 8 TRF-specific pesticides), with all horizontal surfaces accumulating significantly more insecticide than the wall (*p <* 0.0236 for all, Tukey’s test).

By contrast, there were no significant changes in pyrethroid and PBO residues in the bait-only intervention (*p* > 0.3668 for all pesticides studied). Fipronil, the active ingredient in baits, was not detected on these surfaces in any of the bait-treated kitchens either at baseline or one month after bait was applied.

### Economic analysis

The applied material cost of TRFs (in US dollars) ranged from $2.6 to $4.2 per apartment, while gel baits were higher ($11.9 to $16.0, Table 4). However, consumers typically cannot purchase TRFs individually, thus a more realistic comparison would be the realized cost (see Table 4 for equation), which ranged from $7.8 to $12.5, while gel baits ranged from $14.0 to $23.4. Time estimates to complete the interventions were 6 h for TRFs and 2 h for gel baits. TRFs required extensive preparation time (estimated at 1 h), a minimum of 4 h during discharge when residents must vacate the home, and extensive cleanup following deployment (estimated at 1 h). Bait placement was much less time consuming, with maximum time required for application estimated at 1 h, followed by 0.5 h commitments at two-and four-weeks after the initial application.

**Table 4:** Monetary costs and amount of products used for both TRF and gel bait products

Product	Product cost ($US)	Units per package	Unit cost ($US)	Avg. units Used	Applied material cost ($US)	Realized material cost^a^ ($US)
Hot Shot 2	$10.17	3	$3.39	1.0	$3.39	$10.17
Hot Shot 3	$10.17	3	$3.39	1.0	$3.39	$10.17
Raid Deep	$7.79	3	$2.60	1.0	$2.60	$7.79
Raid Fumigator	$12.49	3	$4.16	1.0	$4.16	$12.49
Combat	$6.99	1	$6.99	1.7	$11.88	$13.98
Max Force	$23.43	4	$5.86	3.3	$16.04	$23.43

^a^Realized material cost = Avg. units used / Units per package (rounded up to the nearest whole number), multiplied by the product cost

## Discussion

### TRFs are ineffective at reducing German cockroach infestations

This study provides the first concurrent documentation of the risks associated with TRFs and the ineffectiveness of TRFs at controlling German cockroaches. All TRF products evaluated failed to reduce cockroach populations, providing to our knowledge the first conclusive in-home evidence that these products are inappropriate tools for abatement of German cockroach infestations. Results from population monitoring with traps provided insight into the ineffectiveness of the TRF intervention under “real world” conditions. To test the efficacy of TRFs under ideal conditions, we used laboratory-reared and apartment-collected cockroaches as sentinels in open-top cages during the discharge of TRFs. Whereas all the TRF products killed the insecticide-susceptible laboratory-reared cockroaches, < 38% of the apartment-collected cockroaches died, clearly demonstrating the lack of efficacy of this approach even under ideal conditions.

The overall ineffectiveness of TRFs may be caused by any one or combination of the following factors. First, pyrethroid insecticides are known to be repellent to cockroaches [20–22]. While the sentinel cockroaches could not escape the insecticide deposits from TRFs, the wild cockroaches could behaviorally avoid the insecticide residues. Second, aerosolized particles from TRFs likely failed to reach places where cockroaches normally shelter. We found relatively little insecticide residues on walls near the TRF discharge sites, compared to horizontal surfaces. Since cockroaches are often found under horizontal surfaces (e.g., under the kitchen sink, under countertops, under shelves), they likely avoid the large insecticide deposits on the tops of horizontal surfaces. Finally, and most significantly, extensive and pervasive pyrethroid resistance has evolved in German cockroach populations over the last 3 decades [23–26], rendering even residual spray formulations, which deliver pyrethroids directly to aggregation and foraging sites, ineffective in cockroach abatement [10]. The differential mortality of the insecticide-susceptible laboratory cockroaches and the adjacent wild cockroaches in open-topped cages suggests that the latter might be resistant to pyrethroid insecticides.

### TRFs deposit substantial insecticide residues

All TRF products significantly elevated insecticide residues in homes. With an average increase in insecticide residues of over 600-fold (median 85-fold) for all TRF products, insecticide active ingredients, and horizontal surfaces, it is clear that TRFs can cause significant insecticide contamination in homes. Although the insecticide residues declined after one month, significantly higher levels, relative to baseline, persisted on > 34% of kitchen surfaces. Our results for cypermethrin residues on horizontal surfaces (0.13–0.75 μg/cm^2^, depending on the TRF product and substrate) were comparable to those of Keenan et al. [15] who found 0.07–0.38 μg/cm^2^ after a similar TRF discharge. TRF deposits were also found to settle relatively evenly on all horizontal surfaces (floor: 0.5 m and 1 m from the TRF; countertop; upper cabinet) 4–6 h after discharge, and much less on walls, also shown by Keenan et al. [8]. The high concentrations of pesticide residues in the middle of the kitchen floor, where cockroaches do not aggregate and seldom forage, and low deposits on vertical surfaces where cockroaches often aggregate [7], likely contributed to the ineffectiveness of TRFs.

Considering the extensive deposits of insecticides from the discharge of a single TRF, it is not surprising that many studies, including ours, have found quantifiable “baseline” amounts of a variety of pesticides in homes (including pyrethroids and PBO), without implementing any pest control intervention [9, 17, 27, 28]. A comparison of the floor baseline results from the current study with those reported by Stout et al. [9] reveals some notable trends. First, detection frequencies for all TRF active ingredients were either the same or higher in our study compared with Stout et al. [9]. Our study found higher detection rates for PBO and cypermethrin, while detection rates for permethrin and tetramethrin were within 5%. However, Stout et al. [9] detected fipronil more often. Our MDLs were generally higher than reported by Stout et al. [9], who also sampled surfaces almost 10-fold larger than we did, which could account for our lower incidence of detecting fipronil residues at baseline. For three other insecticides, however, the geometric means were 6- (permethrin), 199- (PBO), and 237-times (cypermethrin) higher in the current study than in Stout et al. [9] (fipronil and tetramethrin were not compared because both were detected at low frequencies in our study). The prevalence of pyrethroid insecticides on kitchen surfaces is particularly remarkable because all our recruited homes were in low-income housing complexes and of lower socio-economic status (SES). In contrast, the homes sampled by Stout et al. [9] represented a random sample of public and private housing units across the U.S. The disparity of these respective studies appears to support previous findings of disproportionate pesticide exposure risks associated with lower SES [29–31]. Future work should focus on better understanding the relationship between pesticide exposure risk and SES.

### Baits: High efficacy and low pesticide residues

In contrast to the TRF formulations, both consumer-based gel bait (Combat) and professional gel bait (Maxforce) were highly effective at reducing the cockroach infestations, as expected based on previous uses of this strategy to mitigate German cockroaches and cockroach-produced allergens [7, 10, 11]. In the current study, baits were used alone, independently of all other integrated pest management (IPM) tactics. Although cockroach reduction was significant among all bait treated homes, populations were not completely eradicated within one month. It is important to note that for complete eradication, inspection and treatment (baiting) would need to continue for longer than the one-month period of this study [32, 33]. Moreover, fipronil (the active ingredient in both bait products) was not detected in swabs in any of the bait-treated apartments, consistent with the findings of Williams et al. [11] that indoor environments under IPM programs involving baits had much lower pesticide residues compared with traditional spray treatments. Furthermore, both TRFs and baits were similarly priced (see Table 4), indicating that cost should not factor into the decision regarding which product to use.

Our effective use of gel baits as a single monitoring-guided intervention, independent of other IPM tactics (e.g., caulking, sanitation, cleaning, trapping), is consistent with an emergent body of literature showing that proper use of baits constitutes the most cost-effective intervention to mitigate the harmful effects of cockroaches and their allergens [reviews: 2, 7]. While more comprehensive IPM approaches e.g. [34, 35, 36] may secure faster and more effective cockroach reductions, the additional costs associated with materials, labor, and administrative matters can make them less practical in many real-world situations.

## Conclusions

It has been over 25 years since Fenske [37] suggested several strategies to mitigate risks associated with indoor pesticide applications, including that “product registrations could be modified or withdrawn for specific applications if an acceptable level of risk cannot be demonstrated.” Although his work focused on active ingredients that in fact have been withdrawn from indoor use, the narrative remains the same – TRF products are depositing large amounts of insecticide residues throughout the home. We now show that TRFs provide no benefits for abatement of German cockroach infestations. Their associated societal costs, lack of observable benefits, and the availability of highly efficacious, affordable, and environmentally sound alternatives, such as baits, challenge the continued registration and persistence of TRF products in the consumer market.

### Study limitations

This study had several limitations, none of which undermine our conclusions. First, this study was limited to a relatively small sample size of homes treated with TRFs, because of IRB concerns with the invasiveness and expected low efficacy of this intervention. Nevertheless, the sample size provided strong statistical power and showed highly significant differences in pesticide residues on hard surfaces in homes and in the efficacy of pest control options. Second, the homes sampled were located within a single city (Raleigh, NC, U.S.A.), potentially raising reservations whether our findings may be generalized globally. It is important to note that the apartments within this city represented a broad range of independent and unrelated low-income apartment complexes, and pervious research showed a general lack of gene flow among cockroaches in different apartment complexes and no pattern of isolation-by-distance [38], suggesting that these five apartment complexes represented independent cockroach populations. Furthermore, our conclusions are generalizable to a global scale because they are supported by (a) residue sampling from 2009 to 2010 in California, with essentially identical active ingredients [15], and (b) surveys documenting pyrethroid resistance in cockroaches on a global scale [23–26].

## Abbreviations

CDC: U.S. Centers for Disease Control
DIY: do-it-yourself
EPA: U.S. Environmental Protection Agency
HUD: U.S. Housing and Urban Development
IPM: Integrated Pest Management
IRB: Institutional Review Board
MDL: Method Detection Limit
NIEHS: U.S. National Institute of Environmental Health Sciences
PBO: Piperonyl Butoxide
SES: Socio Economic Status
TRF: Total Release Fogger
